# Colossal in-plane magnetoresistance ratio of graphene sandwiched with Ni nanostructures

**DOI:** 10.1039/d2ra00957a

**Published:** 2022-05-11

**Authors:** Yusuf Wicaksono, Halimah Harfah, Gagus Ketut Sunnardianto, Muhammad Aziz Majidi, Koichi Kusakabe

**Affiliations:** Graduate School of Engineering Science, Osaka University 1-3 Machikaneyama-cho Toyonaka Osaka 5608531 Japan wicaksono.y@opt.mp.es.osaka-u.ac.jp; Research Center for Quantum Physics, The National Research and Innovation Agency (BRIN) Kawasan Puspiptek Serpong Tangerang Selatan Banten 15314 Indonesia; School of Science, Graduate School of Science, University of Hyogo 3-2-1 Kouto, Kamigori-cho Ako-gun Hyogo 678-1297 Japan; Department of Physics, Faculty of Mathematics and Natural Science, Universitas Indonesia Kampus UI Depok Depok Jawa Barat 16424 Indonesia

## Abstract

In this study, we present a theoretical study on the in-plane conductance of graphene partially sandwiched between Ni(111) nanostructures with a width of ∼12.08 Å. In the sandwiched part, the gapped Dirac cone of the graphene was controlled using a pseudospin by changing the magnetic alignment of the Ni(111) nanostructures. Upon considering the antiparallel configuration of Ni(111) nanostructures, the transmission probability calculation of the in-plane conductance of graphene shows a gap-like transmission at *E* − *E*_F_ = 0.2 and 0.65 eV from the pd-hybridization and controllable Dirac cone of graphene, respectively. In the parallel configuration, the transmission probability calculation showed a profile similar to that of the pristine graphene. High and colossal magnetoresistance ratios of 284% and 3100% were observed at *E* − *E*_F_ = 0.65 eV and 0.2 eV, respectively. Furthermore, a magnetoresistance beyond 3100% was expected at *E* − *E*_F_ = 0.65 eV when the width of the Ni(111) nanostructures on the nanometer scale was considered.

## Abstract 2

In this study, we present a theoretical study on the in-plane conductance of graphene partially sandwiched between Ni(111) nanostructures with a width of ∼12.08 Å.

## Graphical abstract

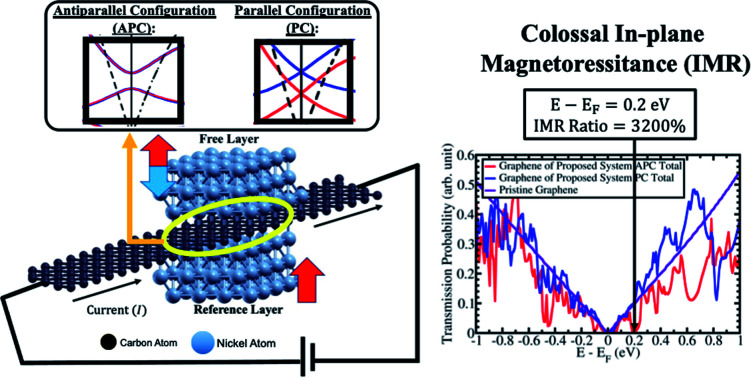

## Introduction

The extraordinary in-plane charge mobility of graphene^1^ makes it an ideal material for applications in micro-electronics^2,3^ and sensing,^4^ due to its novel electronic structure. The equipotential of the C atoms in sublattices A and B of the graphene layer causes a Dirac cone energy band at zero energy. This Dirac cone energy band causes the electrons in graphene to behave peculiarly, having the same velocity and no inertia.^2^ Growing graphene on top of a transition metal substrate enables the sensitive tuning of the Dirac cone energy band. When graphene is placed on a metal substrate, hybridization occurs, breaking the chiral symmetry of graphene and creating a gapped Dirac cone.^5^ Another unique property of graphene is its magnetic response when grown on a ferromagnetic substrate.^6–8^ This induces a magnetic moment in graphene due to charge transfer and has an orientation opposite to that of the ferromagnetic substrate.^8^ Additionally, the weak spin–orbit coupling and long spin scattering length of graphene^9^ make it a prospective material for spintronic devices. Ni(111) surfaces are the most commonly used contacts for studying graphene-based spintronic devices due to their similar structures (smallest lattice mismatch among transition metals) and strong hybridization with graphene.^6,7,10^ Studies show that graphene has served as a bridge between two Ni electrodes^11–13^ because of its long spin relaxation lifetime^9^ and as a tunnel barrier in Ni/graphene/Ni magnetic tunnel junctions (MTJs).^14–23^ Although further results are expected, a new strategy for achieving low resistance and overcoming the absence of a bandgap is necessary to realize the application of graphene in spintronic devices.

Previous studies showed that for a Ni/graphene/Ni heterostructure, the gapped Dirac cone of graphene can be controlled using the magnetic alignment of the Ni slabs.^24^ When the magnetic moments of the upper and lower Ni(111) slabs have an antiparallel configuration (APC), the bandgap at the Dirac cone is open. However, the bandgap is closed in parallel configuration (PC). This unique characteristic is because the most stable arrangement of the Ni/graphene/Ni heterostructure occurs when Ni atoms of the upper and lower Ni(111) slabs at the interfaces hybridize with different graphene sublattices. In other words, Ni atoms from the lower Ni(111) slab hybridized with C atoms in sublattice A (*i.e.*, C_A_), and Ni atoms from the upper Ni(111) slab hybridized with C atoms in sublattice B (*i.e.*, C_B_). Although the C atoms of graphene bond with the Ni atoms, this special hybridization preserves the equipotential between sublattices A and B. Meanwhile, a magnetic moment is induced on the graphene layer by charge transfer from the Ni to C atoms. The induced magnetic moments of the C_A_ and C_B_ atoms depended on the magnetic alignment of the Ni(111) slabs. Moreover, the induced magnetic moments in APC and PC between the C_A_ and C_B_ atoms exhibit antiferromagnetic and ferromagnetic orders, respectively. This implies that the pseudospin between sublattices A and B can be controlled to preserve or break the chiral symmetry, thereby making the gap of the Dirac cone is also controllable. A controllable-gapped Dirac cone help realize a high in-plane magnetoresistance (MR) ratio for graphene.

This study presents a theoretical study on the in-plane conductance and magnetoresistance of graphene partially sandwiched between Ni(111) nanostructures. Here, we investigate the effectiveness of a controllable Dirac cone on graphene conductance to realize a high MR ratio. For that purpose, we proposed a system consisting of Ni(111) nanostructures with a finite size and atomic-scale width of ∼12.08 Å sandwiched the middle part of graphene. Both the APC and PC states of the upper and lower Ni(111) structures are considered. First-principles quantum transport calculations, which coupled density functional theory (DFT) with the nonequilibrium Green's function, were performed. Our calculation results observed a high and colossal in-plane MR ratio of graphene about 284% and 3100%. A higher MR ratio beyond 3100% can be obtained when the width of the Ni(111) structures on a nanometer scale are considered.

## Computational method

**Fig. 1:**
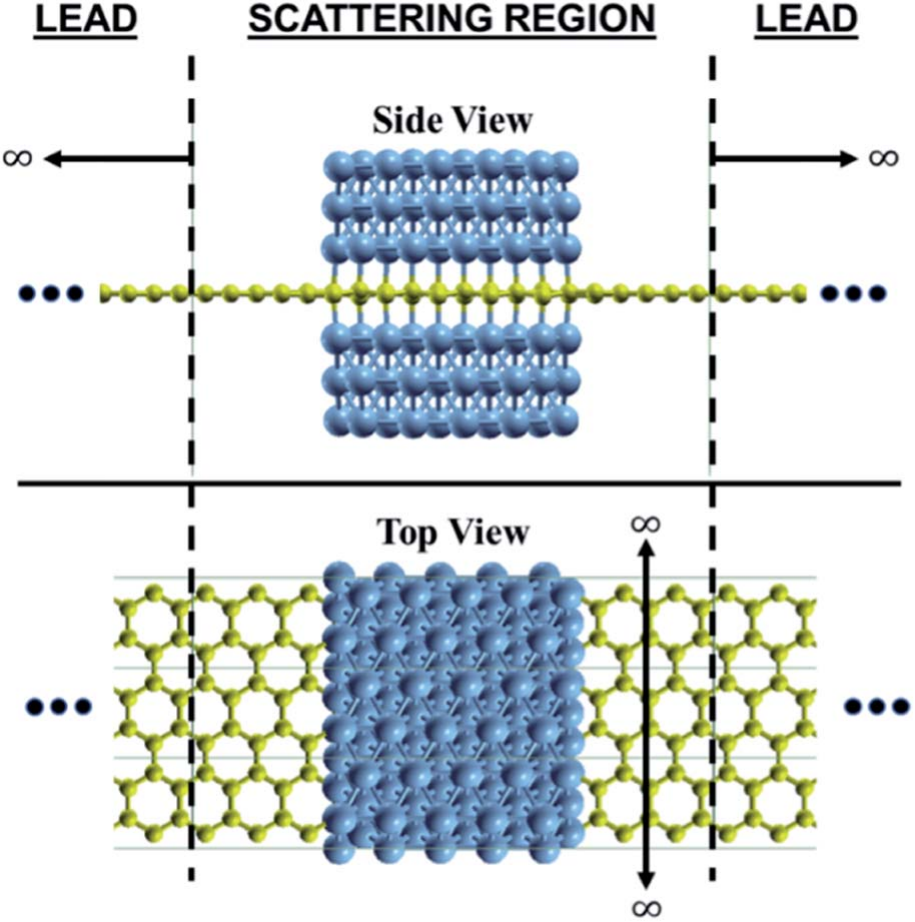
Proposed system of graphene where the middle part is sandwiched by Ni(111) nanostructures. The most stable stacking arrangement from our previous study was considered.^24^ The graphene is used as a buffer and electrode.

In this theoretical study, we considered Ni(111) nanostructures with three Ni atoms layer thickness and an atomic-scale width of ∼12.08 Å, as shown in Fig. 1. The three Ni atoms layer thickness of Ni (111) nanostructures is considered because our previous study suggests that three Ni atoms layer thickness is representable enough for thicker Ni(111) nanostructure by exhibiting same physics at the interface.^24^ The finite size width of ∼12.08 Å for Ni(111) nanostructures was considered for understanding the effectiveness of the in-plane magnetoresistance of graphene created from the Ni/graphene/Ni magnetic junctions. Wider Ni(111) nanostructure, *e.g.*, width in nanometer scale, is expected to exhibit high performance. Here, graphene was used as the buffer layer and electrodes in the calculation. We considered the same stacking arrangement used in the previous study, where Ni atoms at the interfaces of the lower and upper Ni(111) slabs hybridized with C atoms in sublattices A and B, respectively.^24^ Both APC and PC were considered for Ni(111) nanostructures.

A spin-polarized plane-wave-based DFT calculations were performed using the Quantum ESPRESSO package^25^ to obtain the structural equilibrium and spin-charge density properties of the proposed system. Furthermore, we described the electron–ion interaction using a revised Perdew–Burke–Ernzerhof functional for a densely packed solid surface (*i.e.*, PBESol functional)^26^ and ultrasoft pseudopotentials^27^ within the generalized gradient approximation (GGA). From our previous studies, the PBESol pseudopotential is essential to successfully achieve the interlayer distance between Ni slabs and graphene (2D materials in general), which is consistent regardless the thickness of Ni slabs and agrees with experimental study.^24,28,29^ This interlayer distance at the interface is important because it determine the electronic state at the interface. The atomic positions were relaxed, with a total force tolerance of 0.001 eV Å^−1^. A 45 × 45 × 1 Monkhorst–Pack *k*-mesh was used for calculations. First-principles quantum transport calculations, which coupled DFT with the nonequilibrium Green's function, were performed using the Siesta and Transiesta packages^30–34^ to calculate the transport properties at a zero-bias voltage. The spin-dependent current through our proposed system was calculated using the Landauer–Buttiker given by:1
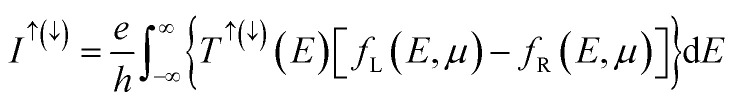
where *f*_L_(*E*,*μ*)(*f*_R_(*E*,*μ*)) is the right (left) moving electron injected from the left (right) lead in the form of the Fermi–Dirac function. *μ*_L_(*μ*_R_) denotes the chemical potentials of the left (right) electrodes. Since zero-bias voltage was considered, thus *μ*_L_ = *μ*_R_ = *E*_F_. In addition, the ballistic transmission *T* as a function of energy *E* is described with respect to the Green's function form as2
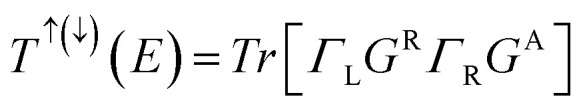
where *Γ*_L_(*Γ*_R_) is the coupling matrix of the left (right) electrode, *G*^R^(*G*^A^) is the retarded (advanced) Green's functions of the central region. Finally, the in-plane MR ratio for graphene was calculated as follows:3
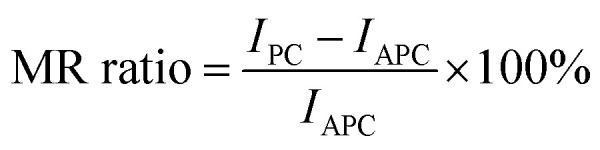


For the transmission probability calculation, the PBESol functional and Troullier–Martins pseudopotential^31^ were used within GGA. A double-zeta plus polarization basis set^35–37^ was employed, and the temperature was set to 300 K. A perpendicular *k*-point of 1 × 901 with respect to the transmission direction was considered to obtain a good accuracy for the transmission probability.

## Results and discussion

### Characteristics of induced magnetic moment of graphene

**Fig. 2:**
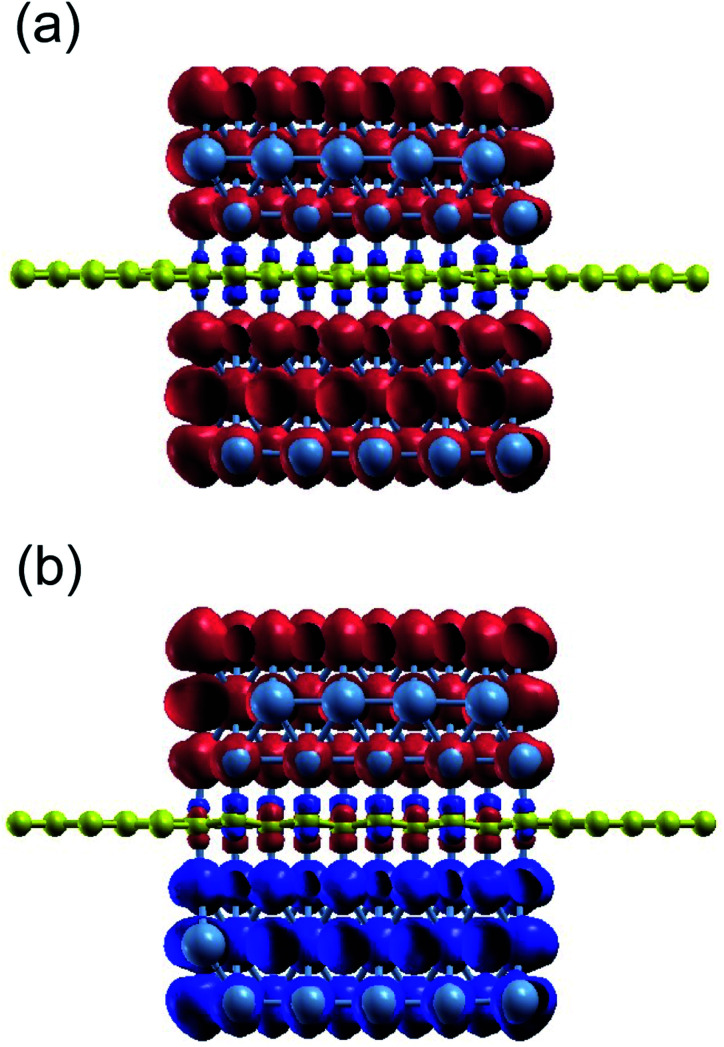
The induced magnetic moment of graphene of the proposed system when the Ni(111) nanostructures are in (a) APC and (b) PC states.

**Fig. 3:**
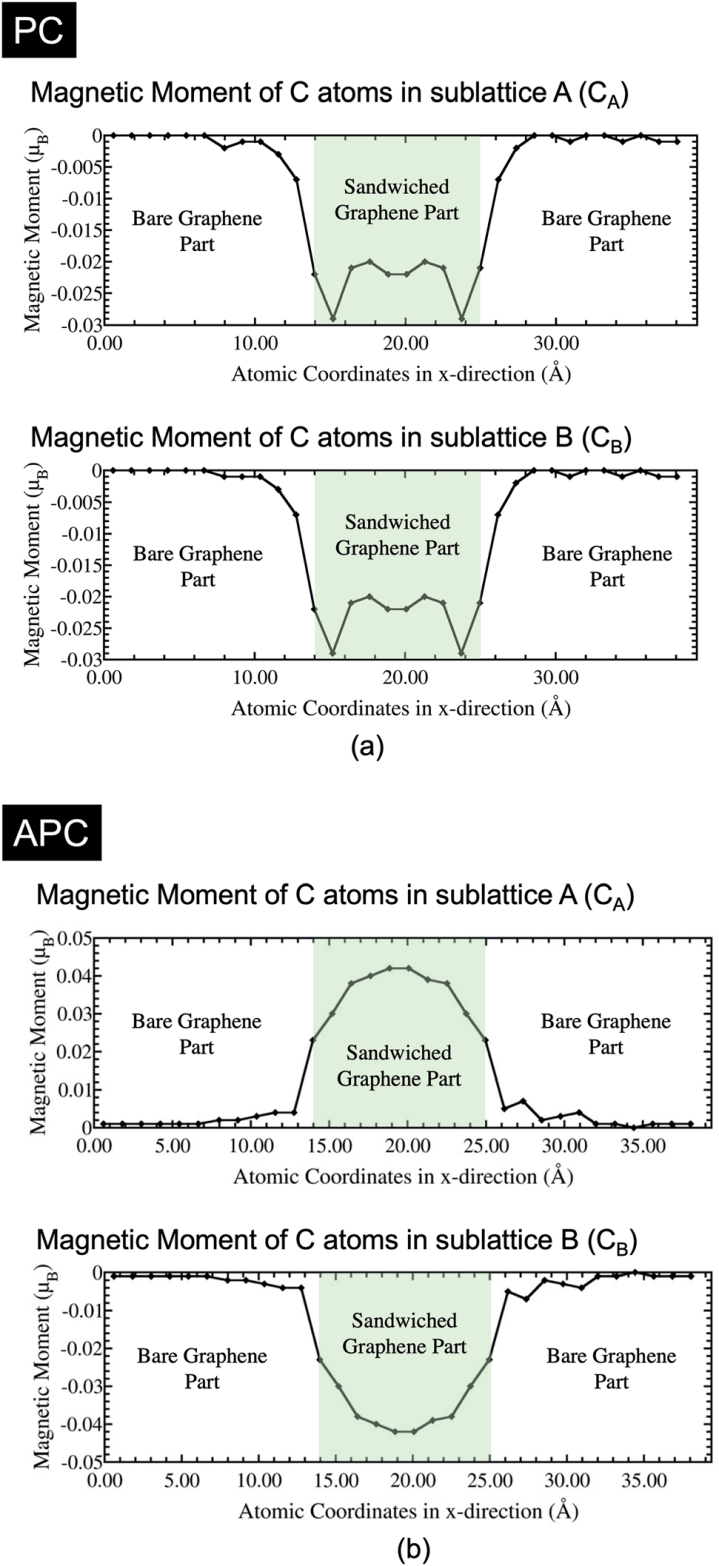
Spin-charge density mapping of the proposed system when the Ni(111) nanostructures are in (a) PC and (b) APC states (red: spin-up charge density; blue: spin-down charge density).

We investigated the characteristics of the induced magnetic moment on graphene, which is sandwiched between the Ni(111) nanostructures. Particularly we focused on the boundaries between the bare and sandwiched graphene. Mapping of the spin-charge density, as shown in Fig. 2, indicates that the magnetic moment was induced only on graphene sandwiched by Ni(111) nanostructures up to the boundaries. However, the details of the induced magnetic moment profile, shown in Fig. 3, indicate that the magnetic moment was damped to a small value on the bare graphene part near the boundaries for a few C atoms before reaching zero. This damping corresponds to wave function matching at the boundaries between sandwiched and bare graphene.

The C atoms of the bare graphene far from the boundaries have no induced magnetic moment, as shown in Fig. 2 and 3. Here, the Dirac cone of the graphene is similar to that of the pristine graphene because the chiral symmetry was preserved: *n*_Aσ_ = *n*_Bσ_ with σ = ↑ or ↓. Meanwhile, the sandwiched graphene has an induced magnetic moment depending on the magnetic alignment of the Ni(111) nanostructures. For the Ni(111) nanostructures in APC state, Fig. 3(a) indicates that the induced magnetic moments on the C_A_ and C_B_ atoms of the sandwiched graphene have an antiferromagnetic order. Thus,4
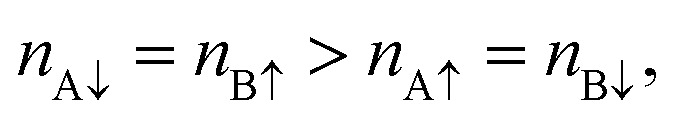
which implies that the chiral symmetry is broken, and the mass gap of the Dirac cone is open. However, for Ni(111) nanostructures in PC state, the induced magnetic moments in the C_A_ and C_B_ atoms of the sandwiched graphene have the same magnetic orientation and almost the same value (Fig. 3(b)). Thus, 5
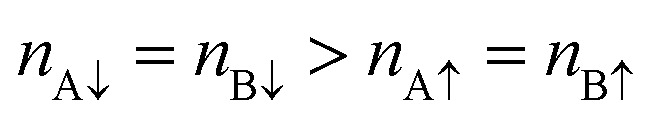


This means that the equipotential between the C_A_ and C_B_ preserves the Dirac cone. However, due to the induced magnetic moment on sandwiched graphene, the Dirac cone on the spin majority channel is lower than that on the spin minority channel, which is a characteristic of ferromagnetic materials.

The magnetic moment characteristics of the C_A_ and C_B_ atoms in the sandwiched graphene agree with the findings of a previous study.^24^ Interestingly, the characteristics of the induced magnetic moments in the sandwiched graphene part were different in the APC and PC states. In the APC state, the amplitude of the induced magnetic moment decreased from the center of the sandwiched graphene to the boundary direction. The magnetic moment amplitude of the C atoms at the center of the sandwiched graphene agrees well with the magnetic moment of the C atoms of Ni/graphene/Ni MTJ (a Ni/graphene/Ni system with periodic boundary conditions on the *x*- and *y*-axes), which is ∼0.04 μ_B_. This means that the decrease in the magnetic moment amplitude along the boundary direction corresponds to the characteristics found in the finite size of the Ni nanostructures. From the structure of the system, this decrease is attributed to the coordination of Ni(111) nanostructures, which compresses their shape toward the center but maintains their bond with C atoms at the interfaces. An edged shape was formed on the Ni(111) nanostructures at the boundaries, which increases the lattice mismatch between the Ni layer at the interfaces and the graphene layer, and in turn, decreases the charge transfer from Ni atoms to C atoms. Thus, the reduced charge transfer from the Ni atoms to the C atoms at the interface decreases the induced magnetic moment on the C atoms. Since the lattice mismatch increases along the direction of the boundary, the induced magnetic moment also decreases along the same direction. However, the profile of the induced magnetic moment on sandwiched graphene is unique in PC. The induced magnetic moment initially decreases from the center of the sandwich to the boundaries. However, it then increases even higher than that of the center part before finally decreasing again near the boundaries. The magnetic moment of the C atoms in the center of the sandwiched graphene was approximately equal to the induced magnetic moment of the C atoms of the Ni/graphene/Ni MTJ, which was ∼0.022 μ_B_. Furthermore, the decrease in the induced magnetic moment on the neighboring C atoms, which shifted from the center of the sandwich graphene to the boundary, corresponds to the increasing lattice mismatch, similar to the case of APC. Additionally, the amplitude of the magnetic moment on the C atoms increased even more than that in the center part at the boundaries due to the reduction in the antiferromagnetic configuration between sublattices A and B near the boundary. In the periodic system of Ni/graphene/Ni MTJ, we obtained higher amplitude of the induced magnetic moment of C atoms in APC is higher than that in PC^24^ since the carbon atoms of sublattices A and B in graphene had to have an antiferromagnetic (AFM) configuration because of the half-filled p_*z*_-orbital and Pauli's exclusion principle. This rule is often found in organic molecules in sp^2^-hybridization or magnetic alternant hydrocarbon systems.^38^ The contribution of the antiferromagnetic alignment between the hybridized C and Ni atoms near the boundaries is more dominant than that of the AFM alignment between the C atom sublattices A and B, leading to the increased induced magnetic moment. Finally, at the boundaries, the induced magnetic moment of the C atoms in the sandwiched part decreases again, indicating increased lattice mismatch.

These changes in the induced magnetic moment on sandwiched graphene affect the Dirac cone and mass-gapped Dirac-cone characteristics. For the Ni(111) nanostructures in the APC, the decreasing induced magnetic moment of the sandwiched graphene toward the boundary reduces the size of the mass-gapped Dirac cone. This implies that the Dirac cone mass-gapped is smaller at the boundaries of the sandwiched graphene than at the center. For Ni(111) nanostructures in the PC state, the changes in the induced magnetic moment affect the size of the Stoner gap between the spin majority and spin minority channels. These characteristics are discussed in detail in the next section.

### Colossal in-plane magnetoresistance of graphene

**Fig. 4:**
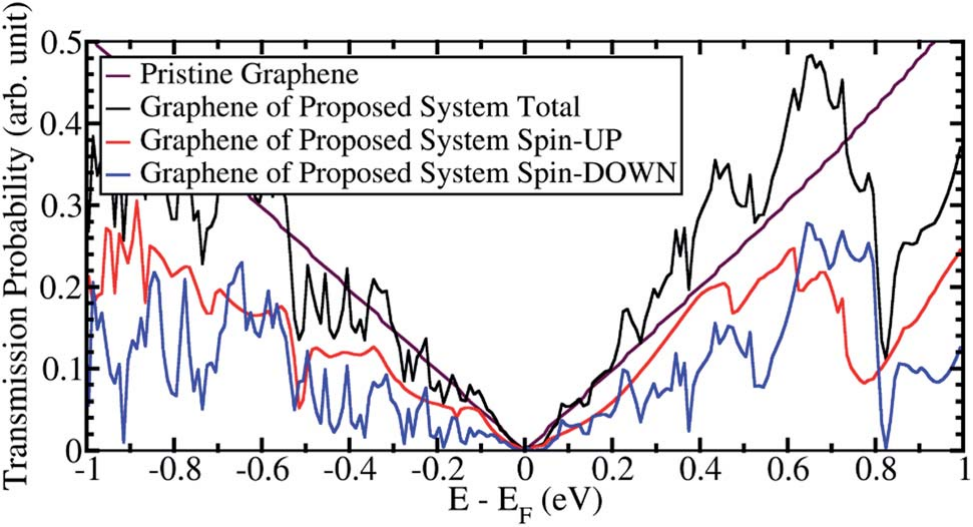
The transmission probability of graphene from the proposed system when Ni(111) nanostructures in PC state.

**Fig. 5:**
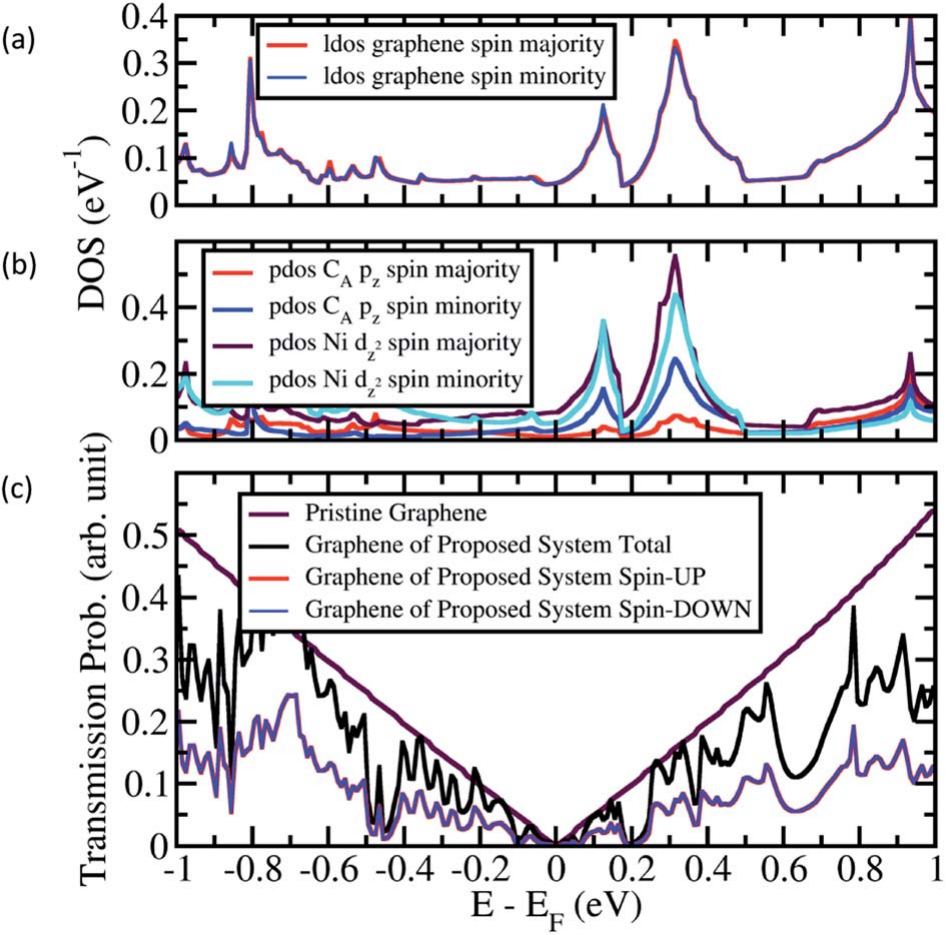
(a) The local density of states (LDOS) of graphene in Ni/graphene/Ni MTJ, (b) projected density of states (PDOS) of C_A_ and Ni at interface hybridized with C_A_, and (c) the transmission probability of graphene from the proposed system when Ni(111) nanostructure in APC state.

Fig. 4 and 5(c) show the transmission probability of electrons through sandwiched graphene from our proposed system when the Ni(111) nanostructures are in the PC and APC states, respectively. When the Ni(111) nanostructures were in the PC state, the transmission probability profile for the proposed system is similar to that of the pristine graphene, as shown in Fig. 4. The typical transmission probability of the pristine graphene originates from the Dirac cone of graphene, where the transmission probability results in zero conductance at the Fermi energy and increases linearly with energy. The slight increase in the total transmission probability comes from spin-up electrons that transmit through graphene and Ni(111) nanostructures. Thus, spin-up electrons have a higher transmission probability than the spin-down electrons.

Further, a profile similar to that of the pristine graphene was observed when the Ni (111) nanostructures were in the APC state, as shown in Fig. 5(c). However, a unique gap-like transmission probability was found at *E* − *E*_F_ = 0.18–0.22 eV and 0.58–0.78 eV, with its lowest transmission probability at *E* − *E*_F_ = 0.2 and 0.65 eV, respectively, to understand the origin of the gap-like transmission, local density of states (LDOS) and projected density of states (PDOS) calculations were performed on the Ni/graphene/Ni MTJ, which represents the sandwiched part of the proposed system. The LDOS of graphene, shown in Fig. 5(a), suggests that the Dirac-cone-like density of states (DOS) which was observed at *E* − *E*_F_ = 0.2 eV corresponds to the gap-like transmission probability found at *E* − *E*_F_ = 0.2 eV. This relation was observed in the spin majority and minority channel. This Dirac-cone-like DOS is originated from the hybridization between d^2^_*z*_-orbital of Ni atoms at the interface and p_*z*_-orbital of C atoms (Fig. 5(b)). The Dirac-cone-like DOS profile at *E* − *E*_F_ = 0.2 eV, which was observed in LDOS of graphene, was also found in both the PDOS of p_*z*_-orbital of C_A_ atoms and d^2^_*z*_-orbital of Ni atoms below C_A_ atoms, implying the characteristics of hybridization (Fig. 5(b)).

The gap-like transmission probability at *E* − *E*_F_ = 0.65 eV results from the opening mass-gapped Dirac cone of graphene (Fig. 5(a)). However, compared to the mass-gapped Dirac cone of graphene shown in Fig. 5(a), the width of the transmission probability gap of the proposed system is smaller. Furthermore, the transmission probability at *E* − *E*_F_ = 0.65 eV is considerably high, unlike the LDOS of graphene in Fig. 5(a); the DOS at *E* − *E*_F_ = 0.65 eV is almost zero similar to DOS at *E* − *E*_F_ = 0.2 eV. This difference is because the induced magnetic moment differs between the C atoms at the boundaries and center of the sandwiched graphene, thereby leading to different mass-gapped size and a parabolic transmission probability gap with smaller gap size. Moreover, the small width of Ni (111) nanostructure does not optimize the induced magnetic moment on graphene and a constant value is not achieved, thereby resulting considerably high conductance at *E* − *E*_F_ = 0.65 eV. However, this also implies that, when a width of Ni(111) nanostructures is considered in the nanometer scale, the transmission probability at *E* − *E*_F_ = 0.65 eV will be as low as at *E* − *E*_F_ = 0.2 eV.

**Fig. 6:**
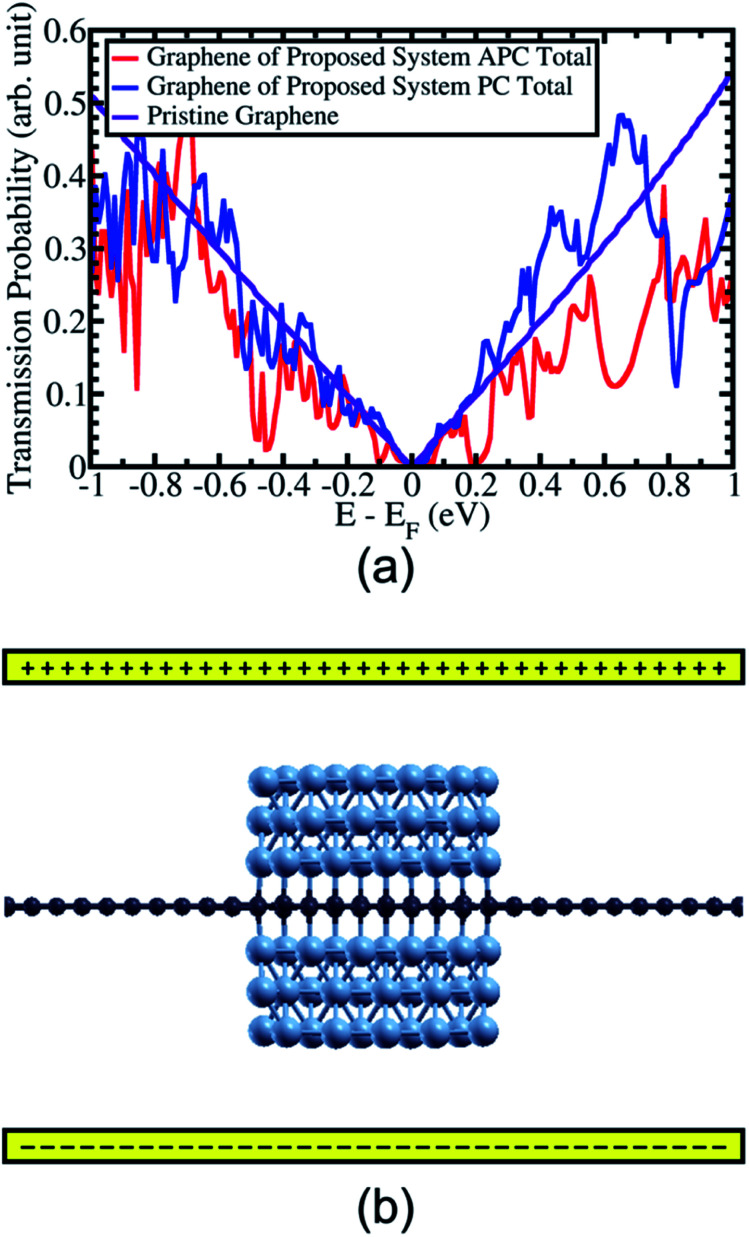
(a) The comparison of transmission probability of graphene from the proposed system between APC and PC states. (b) The configuration of gate voltage on the proposed system to shifting the Fermi energy.

By comparing the transmission probability of electrons of the Ni(111) nanostructures in APC and PC states, a high and colossal in-plane MR ratio of up to 3100% and 284% was observed at *E* − *E*_F_ = 0.2 and 0.65 eV, respectively(Fig. 6(a)). Applying a gate voltage to the proposed system shifts the Fermi energy^39,40^ to *E* − *E*_F_ = 0.2 eV resulting in a colossal in-plane MR ratio (Fig. 6(b)). Interestingly, this high in-plane MR ratio can be achieved just by sandwiching graphene with Ni(111) nanostructures with an atom-scale width of ∼12.08 Å. By increasing the width of Ni(111) nanostructure to the nanometer scale, a colossal MR ratio of >3100% can be expected at *E* − *E*_F_ = 0.65 eV because in APC state the gap-like transmission will be nearly zero similar to that at *E* − *E*_F_ = 0.2 eV; however, the transmission probability is higher than that at *E* − *E*_F_ = 0.2 eV when PC state is considered.

## Conclusions

In this study, we investigated the in-plane conductance of graphene, where the Ni(111) nanostructures with a width of ∼12.08 Å is sandwiched in the center of the graphene. Both the APC and PC states of the Ni(111) nanostructures were considered. The induced magnetic moment was observed on the sandwiched graphene and could be controlled using the magnetic alignment of the Ni(111) nanostructures. When APC (PC) state is considered, we observed that the carbon atoms of sublattices A and B of the sandwiched graphene had an antiferromagnetic (ferromagnetic) order. Both spin configurations lead to a controllable mass-gapped Dirac cone in the sandwiched graphene. The mass-gapped Dirac cone is open (closed) for APC (PC) state due to the modulated (equi-) potential between the C_A_ and C_B_ of the sandwiched graphene.

When the Ni(111) nanostructures were in the PC state, the transmission probability of the proposed system produces a profile similar to that of the pristine graphene. Furthermore, a slight increase in the total transmission probability was observed from spin-up electrons that transmit through graphene and the Ni(111) nanostructures. Thus, spin-up electrons have a higher transmission probability than the spin-down electrons.

However, for the Ni (111) nanostructures in the APC state, a unique gap-like transmission probability was observed from *E* − *E*_F_ = 0.18–0.22 eV and 0.58–0.78 eV with the lowest transmission probability at *E* − *E*_F_ = 0.2 and 0.65 eV, respectively. The gap-like transmission probability at *E* − *E*_F_ = 0.2 eV comes from the Dirac-cone-like DOS shown in the local density of sandwiched graphene due to the hybridization property between the d^2^_*z*_-orbital of Ni atoms at the interface and p_*z*_-orbital of C atoms. On the other hand, the gap-like transmission probability at *E* − *E*_F_ = 0.65 eV comes from the opening mass-gapped Dirac cone of graphene. However, the gap-like transmission probability was smaller than that found in the LDOS of graphene in Ni/graphene/Ni MTJ. This is because the induced magnetic moment decayed from the center part of the sandwiched graphene toward the boundary, leading to a parabolic shape for the transmission probability gap.

Finally, high and colossal in-plane MR ratios of up to 3100% and 284% were observed at *E* − *E*_F_ = 0.2 and 0.65 eV. By applying a gate voltage, the Fermi energy can be controlled resulting in a colossal in-plane MR ratio. Furthermore, by increasing the width of the Ni(111) nanostructure to the nanometer scale, a colossal MR ratio > 3100% can be expected because the gap-like transmission is nearly zero at *E* − *E*_F_ = 0.65 eV in the APC state but having higher the transmission probability than that at *E* − *E*_F_ = 0.2 eV in the PC state.

## Author contributions

Conceptualization was performed by Y. W., K. K., H. H., and G. K. S. performed computer simulations of the atomic layered systems and M. A. M. conducted the theory of atomic layered materials. Y. W. determined the equilibrium structure of the proposed system and performed spin-charge density mapping and transmission probability analysis on the proposed system. YW wrote the original draft of the manuscript. All the authors have reviewed the manuscript. The manuscript was written through the contributions of all authors. All the authors approved the final version of the manuscript.

## Conflicts of interest

There are no conflicts of interest to declare.

## Supplementary Material
